# Redescription of *Corydoras undulatus* Regan, 1912 (Siluriformes: Callichthyidae), with comments on the identity of *Corydoras latus* Pearson, 1924

**DOI:** 10.1371/journal.pone.0211352

**Published:** 2019-01-28

**Authors:** Alessandra Bono, Luiz Fernando Caserta Tencatt, Felipe Alonso, Pablo Lehmann A.

**Affiliations:** 1 Laboratório de Ictiologia, Universidade do Vale do Rio dos Sinos, São Leopoldo, Rio Grande do Sul, Brazil; 2 Universidade Estadual de Mato Grosso do Sul, Unidade Universitária de Coxim, Rua General Mendes de Moraes, Coxim, Mato Grosso do Sul, Brazil; 3 CONICET- IBIGEO, Instituto de Bio y Geociencias del NOA, Rosario de Lerma, Salta, Argentina; Southwest University, CHINA

## Abstract

A redescription of *Corydoras undulatus* Regan, 1912 is presented. The original description of *C*. *undulatus* is very succinct, as is its diagnosis, which is based only on external morphology. Additional information in the scientific literature on this species is scarce. Specimens from the distribution area of this species were analyzed; Paraná and Paraguay river basins in Argentina, Uruguay river basin in Argentina, Brazil and Uruguay, and the Laguna dos Patos system in Brazil. Morphological analysis, principal component analysis (PCA), meristic comparison and osteological description were performed. *Corydoras undulatus* can be distinguished from its congeners mainly by having the following combination of characters: mesethmoid short, with anterior tip short, smaller than 50% of the entire bone length; posterior margin of the pectoral-fin spine with nearly all serrations directed towards origin of spine; pectoral-fin spine with conical serrations; and its peculiar color pattern. The analysis of the material from the different basins did not indicate relevant morphological differences, suggesting that the species presents a wide distribution in La Plata and Laguna dos Patos drainages. The shared geographic distribution between these two systems is also present in other fish species. The current work presents data about the type locality, taxonomy, osteology, distribution and ontogenetic variation of color pattern in *C*. *undulatus*. Comments on the identity of a very similar congener, *Corydoras latus*, will also be provided.

## Introduction

*Corydoras* Lacépède, 1803 comprises the majority of species of the Corydoradinae, and represents the genus with the greatest species-rich among the Siluriformes. With 174 valid species, 19 described in the last ten years, besides presenting many species not yet described [1,2].

An important comprehensive study on *Corydoras* species from the Suriname was carried out by Nijssen [3] in which *Corydoras* was divided in nine groups of species based mainly on the color, morphometric and meristic patterns. *Corydoras undulatus* Regan, 1912 was included along with 15 other species in the "Barbatus-group" based on brownish spots on dorsolateral body plates, alternating more or less with the ventrolateral plates.

Ten years later, Nijssen & Isbrücker [4] present a review of the *Corydoras* listing the measurements and counts of the 115 species analyzed. In this work, these authors reformulated the groupings originally proposed by Nijssen [3], reducing them from nine to five. *Corydoras undulatus* was included into the "*Corydoras elegans*-group" with seven other species: *C*. *elegans* Steindachner, 1877 (Amazon River at Tefé, Amazonas, Brazil); *C*. *hastatus* Eigenmann & Eigenmann, 1888 (Villa Bella [= Parintins, 2°38'S 56°45'W], Amazonas, Brazil); *C*. *latus* Pearson, 1924 (Lagoons, Lago Rojoagua [Rogoagua], Río Beni basin, Upper Amazon system, Beni, Bolivia); *C*. *guapore* Knaack, 1961 (main stream of upper Rio Guaporé, Rondônia, Brazil); *C*. *pygmaeus* Knaack, 1966 (near Calama, 8°05'S 62°52'W, along Rio Madeira near mouth of Rio Jipiraná [= Machado River], Rondônia State, Brazil); *C*. *nanus* Nijssen & Isbrücker, 1967 (little tributaries of Gran-Rio between Ligoria [= Ligolio] and Awaradam Falls, Brokopondo, Suriname); *C*. *gracilis* Nijssen & Isbrücker, 1976 (Rio Jauna [= River Juma] at Trans-Amazonica highway, about 6°09'S, 59°55'W, tributary of Rio Aripuaña, Amazonas, Brazil [corrected locality]).

*Corydoras undulatus* was described by Charles Tate Regan in 1912 [5] with only 3 specimens. The holotype, catalog number: BMNH 1912.7.10.5, 43.9 mm SL, female, was donated by Dr. Willy Georg Wolterstorff. The two paratypes, catalog number: BMHN 1909.9.28.3–4, 26.7 mm SL and 27.8 mm SL, were donated by Johann Paul Arnold. Both lots are indicated by Regan as coming from “La Plata” [5]. The holotype and paratypes are deposited in the collection of the Natural History Museum in London. The original diagnosis is based only on external morphology and additional information in the scientific literature on the species is scarce.

This species presents a wide geographic distribution in the Río de La Plata basin and coastal rivers of Southern Brazil. In the Uruguay Republic the species was first recorded by Nion *et al*. [6], but no collection locality or voucher specimens were presented. Teixeira De Mello *et al*. [7] mention the presence of this species in that country in the “Uruguay River basin north of the Rio Negro”, but still no concrete localities or collection specimens are presented. Here we present also the first confirmed record of this species with collection material from the Uruguay Republic. Also, there are lots of this species deposited in the Swedish Museum of Natural History with collecting localities in the Paraguay River drainage and herein we add three more localities for this basin in Argentina.

Whereas the original description of *Corydoras undulatus* contains limited information on morphological characters and, mainly, osteological characters, having been based on only three specimens; here we provide a redescription of *C*. *undulatus*, detailing its geographic distribution area, Laguna dos Patos system, Paraná, Uruguay and Paraguay basins. Additionally, a brief discussion on the dubious identity of one of its most similar congeners, *C*. *latus*, confirmed herein as a member of the “*C*. *elegans* group”, is also provided.

## Material and methods

The live specimens were anesthetized in a Eugenol solution dissolved in ethyl alcohol in 1:9 ratio (clove oil: ethyl alcohol), and this solution was then diluted with water in order to obtain concentrations of 0.20 mL of clove oil per 500 mL of water. To follow, specimens were fixed in 10% formalin, and then transferred to 70% ethanol. This protocol has been approved by the commission of ethical use of animals of Universidade do Vale do Rio dos Sinos (CEUA/UNISINOS) which considerers animal welfare laws. The morphological measurements were obtained using a precision digital caliper in tenths of millimeters. Meristic and morphometric data were taken following Reis [8] with modification of Tencatt *et al*. [9]. Morphometric measures are presented as percentages of standard length (SL) and head length (HL). Based on the morphometric data, Principal Component Analysis (PCA) was performed with PAST 1.0 statistical program. Some specimens were cleared and stained (c&s) following Taylor & Van Dyke [10]. The homology of the barbels follows Britto & Lima [11]. The osteological terminology was based on Reis [12], except for the parieto-supraoccipital instead of the supraoccipital [13], the pterotic compound instead of the supracleithrum [14] and the scapulocoracoid instead of the coracoid [15]. The nomenclature of the lateral-sensory canals and the preopercular pores is in agreement with Schaefer & Aquino [16] and Schaefer [17], respectively. The supra-preopercle *sensu* Huysentruyt & Adriaens [18] was treated here as a part of the hyomandibula. Vertebral counts include only free vertebrae, with the compound caudal centra (preural 1+ ural 1) counted as a single element. The stripes were counted as in Tencatt & Ohara [19].

Comparative data of *Corydoras bilineatus*; *C*. *latus*; *C*. *mamore* Knaack, 2002; *C*. *nanus*; *C*. *nijsseni* Sands, 1989 and *C*. *paucerna* Knaack, 2004 were obtained through their original descriptions and/or high resolution photographs of type-specimens available from Morris et al. [20] and from the California Academy of Sciences Ichthyology Primary Types Imagebase, available at http://researcharchive.calacademy.org/research/ichthyology/types/index.asp.

The numbers in parentheses represent the total number of specimens showing the respective count and the asterisks indicate the counts of the holotype. Institutional abbreviation follow Sabaj [21], except UNICTIO (Laboratório de Ictiologia, Universidade do Vale do Rio dos Sinos, UNISINOS, São Leopoldo); MZU (Museu Zoológico da Unisinos, Universidade do Vale do Rio dos Sinos, UNISINOS, São Leopoldo); IBIGEO (Instituto de Bio y Geociencias del NOA—Argentina).

## Results

### *Corydoras undulatus* Regan, 1912 (Figs 1–3)

*Corydoras undulatus* Regan, 1912: 217 (original description; type-locality: La Plata, Buenos Aires, Argentina).–Nijssen & Isbrücker, 1980: 214, 215 (listed; measurements and counts of the holotype on table IX).–Malabarba, 1989: 147 (listed).–Isbrücker 2001: 237 (listed).–Knaack 2002: 52, 54 (listed as comparative material; figure of a living specimen).–Reis *et al*., 2003: 304 (listed).–López *et al*., 2003: 43 (description; figure of a living specimen).–Casciotta *et al*., 2005: 168, 233 (fig 89).–Menni, 2004: 84, 111, 189 (Tables 11.2 and 32.1).–Almirón *et al*., 2008: 152 (fig).–Teixeira de Melo, 2011: 95 (fig).–Ferraris, 2007: 126 (listed).–Litz & Koerber, 2014: 23 (listed).–Mirande & Koerber, 2015: 40 (listed).

**Fig 1:**
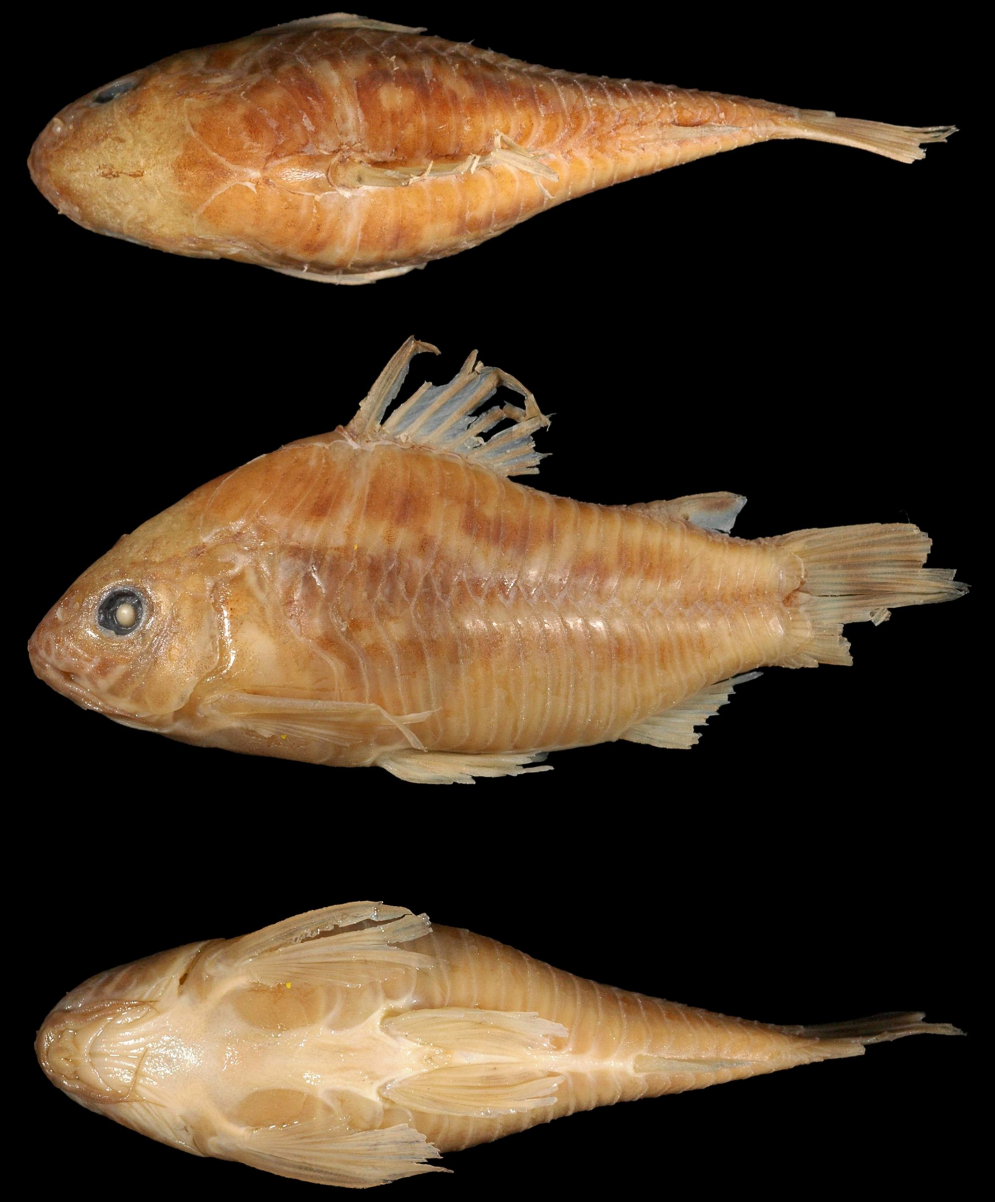
*Corydoras undulatus*, holotype. BMNH 1912.7.10.5, 43.9 mm SL, “La Plata” locality. Donor Dr. Willy Georg Wolterstorff. Dorsal, lateral and ventral view. “The Trustees of the Natural History Museum, London”. (Photograph and credit by Mark Allen, All Catfish Species Project).

**Fig 2:**
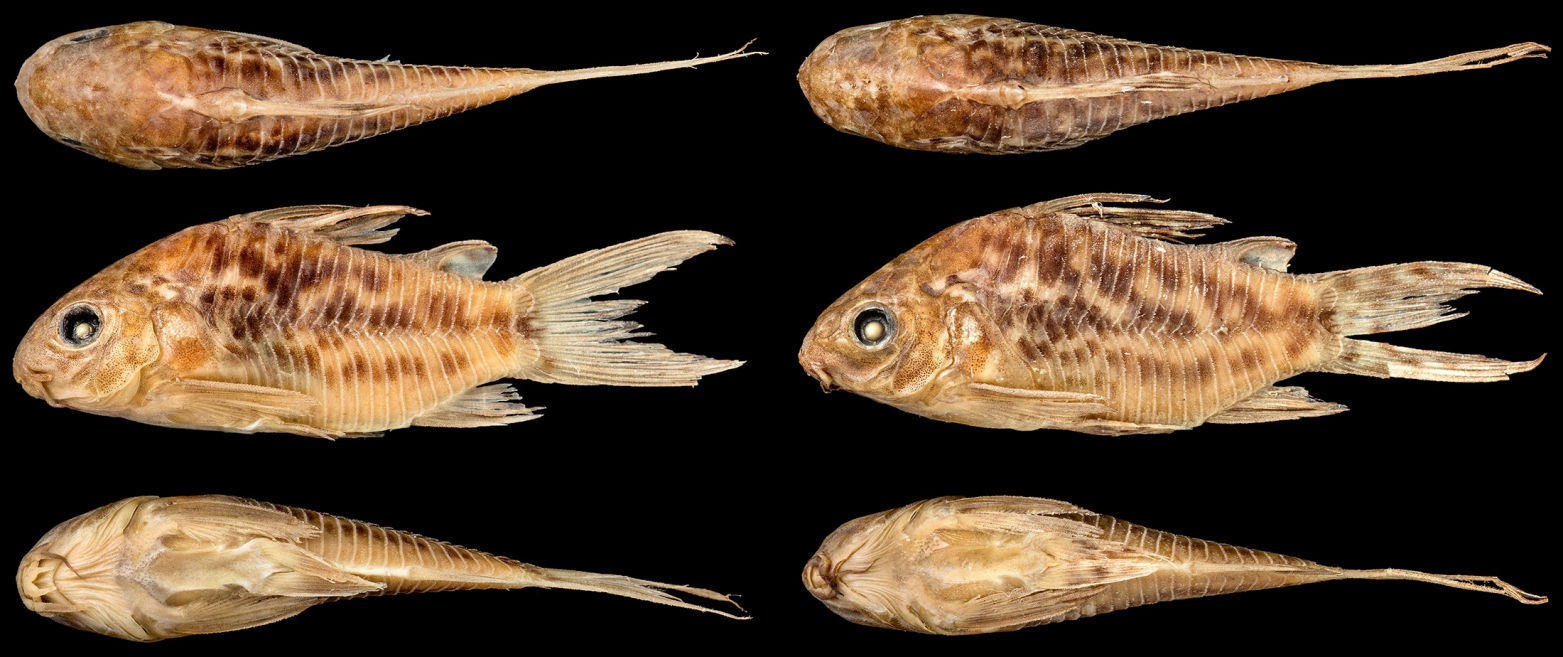
*Corydoras undulatus*, paratypes. BMNH 1909.9.28.3–4, 26.7 mm SL and 27.8 mm SL, “La Plata” locality. Donated by Johann Paul Arnold. Dorsal, lateral and ventral view. “The Trustees of the Natural History Museum, London”. (Photograph and credit by Mark Allen, All Catfish Species Project).

**Fig 3:**
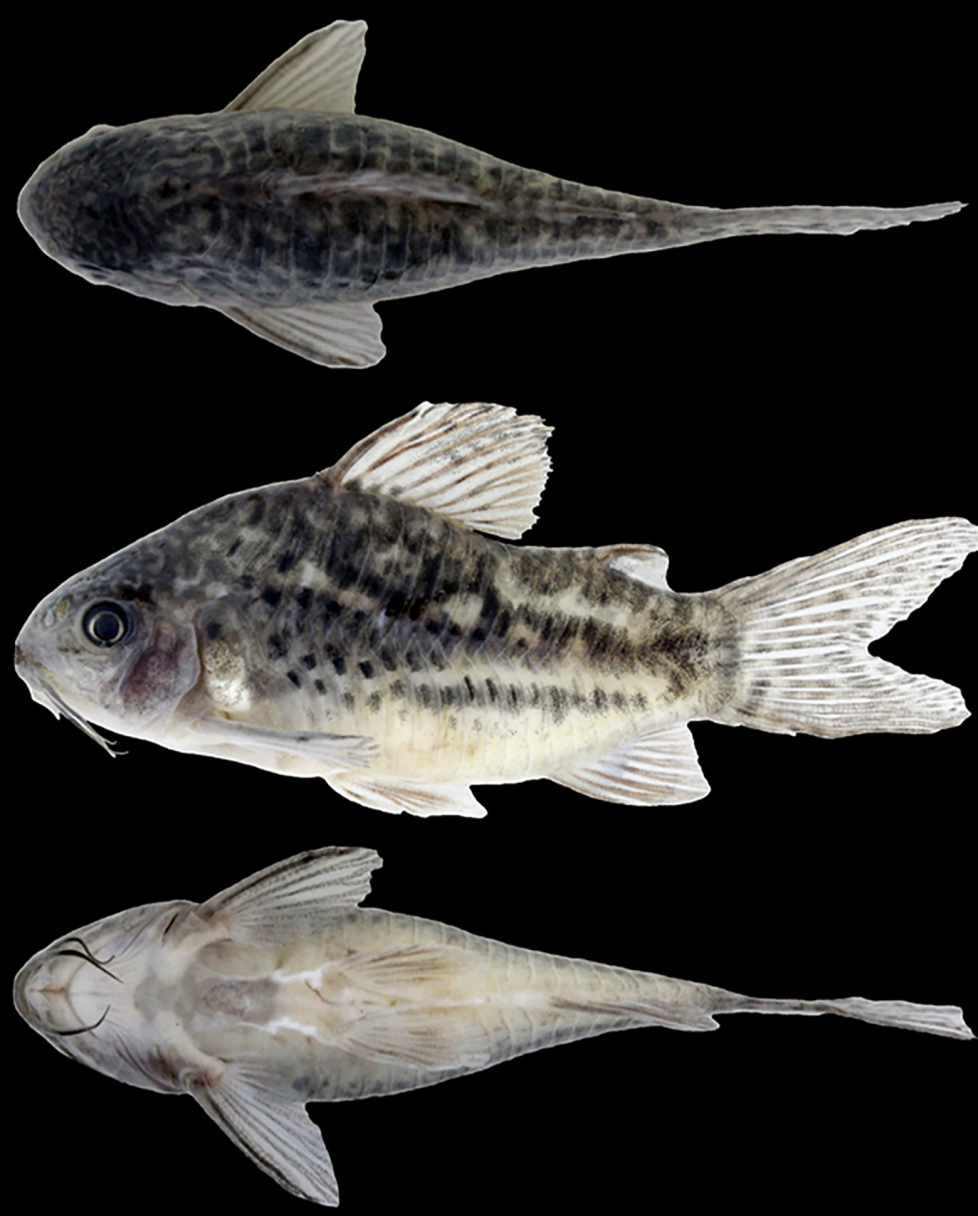
*Corydoras undulatus*. IBIGEO-I 451, 37.4 mm SL, from San Lorenzo stream, Paraná River basin, at Entre Ríos Province in Argentina. Dorsal, lateral and ventral view.

### Diagnosis

*Corydoras undulatus* can be distinguished from its congeners, with exception of *C*. *bilineatus*, *C*. *elegans*, *C*. *gracilis*, *C*. *guapore*, *C*. *latus*, *C*. *mamore*, *C*. *nanus*, *C*. *napoensis* Nijssen & Isbrücker, 1986, *C*. *nijsseni*, and C. *paucerna*, by having the following combination of features: mesethmoid short, with anterior tip short, smaller than 50% of entire bone length (*vs*. long, with anterior tip larger than 50% of entire bone length); posterior margin of pectoral-fin spine with nearly all serrations directed towards origin of spine (*vs*. all or nearly all serrations perpendicularly inserted or directed towards tip of spine); pectoral-fin spine with conical serrations (*vs*. laminar). It differs from *C*. *bilineatus*, *C*. *elegans*, *C*. *gracilis*, *C*. *guapore*, *C*. *latus*, *C*. *mamore*, *C*. *nanus*, *C*. *napoensis* and *C*. *paucerna* by having flanks with relatively large dark brown or black markings, forming a marbled or somewhat anastomosed pattern at least on anterior portion of flanks; blotches variably forming up to three irregular and/or intermittent longitudinal bands; first band, if present, along dorsolateral body plates, second, if present, along midline of flank, and third, if present, along ventrolateral body plates; third band more regular and continuous in some specimens (*vs*. generally with clearly more regular and distinct pattern of longitudinal stripes on flanks, which alternate transversely a dark brown or black stripe with a brownish yellow stripe; marbled or somewhat anastomosed pattern, if present, restricted to anterior portion of body in *C*. *bilineatus* and *C*. *napoensis*; dorsal portion of flank with a long, continuous, regular, longitudinal dark brown or black stripe, which runs in parallel to body dorsal profile, extending at least from anterior portion of dorsal-fin base to posterior portion of caudal peduncle; stripe generally with broad anterior portion, becoming narrower towards its posterior portion; dorsally and ventrally bordered by scarcely spotted regions, which often form longitudinal brownish yellow longitudinal bands; region midline of flank and ventrolateral body plates with dark brown or black spots, which can be aligned in longitudinal rows; midline of flank variably with a slender longitudinal stripe in *C*. *elegans*; dorsal portion of flank with a long, arched, continuous black stripe, which runs parallel to body dorsal profile, extending at least from corner of mouth region to posterior portion of caudal peduncle, dorsally and ventrally bordered by scarcely spotted regions, which often form longitudinal brownish yellow longitudinal bands; ventrolateral body plates with small dark brown or black blotches, variably aligned in longitudinal rows, in *C*. *gracilis*; flank mostly covered by small dark brown or black blotches, not forming a marbled or anastomosed pattern, with a large roundish dark brown or black blotch covering caudal peduncle region in *C*. *guapore*; ventral portion of dorsolateral body plates and dorsal portion of ventrolateral body plates with a longitudinal row of brownish yellow small roundish areas, more evident on flanks anterior half; remaining areas of dorsolateral body plates with conspicuous concentration of dark brown or black chromatophores, more evident on flanks anterior half; dorsal half of ventrolateral body plates, except for small brownish yellow roundish areas, with conspicuous concentration of dark brown or black chromatophores in *C*. *latus*; with relatively small dark brown or black blotches, not forming a marbled or anastomosed pattern in *C*. *mamore* and *C*. *paucerna*; dorsal portion of flank with a long, continuous, regular, longitudinal dark brown or black stripe, which runs in parallel to body dorsal profile, extending at least from dorsal-fin base anterior portion to caudal peduncle posterior portion; dorsally and ventrally bordered by scarcely spotted regions, which often form longitudinal brownish yellow longitudinal bands; ventral portion of dorsolateral body plates and dorsal portion of ventrolateral body plates with a series of longitudinally aligned dark brown or black spots, which merge at dorsal-fin region and form a stripe, that can range from narrow to broad; ventrolateral body plates with dark brown or black blotches, generally longitudinally aligned in *C*. *nanus*); from *C*. *nijsseni* by absence of a conspicuous concentration of dark brown or black chromatophores on eyes region, transversally disposed and forming a mask-like blotch (*vs*. presence).

### Description

Morphometric data shown in Table 1. Head slightly compressed in dorsal view, with convex dorsal profile in lateral view. Head roughly triangular or somewhat pentagonal in dorsal view. Snout short and rounded; strongly reduced in some specimens. Head dorsal profile convex from snout tip to anterior nostrils; ascending slightly convex or nearly straight from this point to dorsal-fin origin. Profile slightly convex along dorsal-fin base. Body profile posterior to dorsal fin slightly concave to adipose-fin spine; concave from this point to caudal-fin base. Ventral profile of body slightly convex from isthmus to pelvic-fin origin; region of gill opening slightly concave in some specimens; slightly convex or nearly straight from this point to anal-fin origin; concave until caudal-fin base. Body roughly elliptical in cross section at pectoral girdle, gradually becoming more compressed towards caudal fin.

**Table 1:** Morphometric data of the holotype and 157 non-type specimens of *Corydoras undulatus*.

	Holotype	Min	Max	Mean	SD
Standard length (mm)	41.8	15.7	46.8	30.5	-
**Percents of Standard Length**					
Depth of body	45.9	36.4	45.9	41.1	1.6
Predorsal distance	52.4	47.4	55.9	51.1	1.8
Prepelvic distance	49.8	45.2	52.5	48.7	1.5
Preanal distance	81.6	72.2	81.7	77.2	1.8
Preadipose distance	90.9	80.1	90.9	83.8	1.5
Length of dorsal spine	15.6	15.6	24.8	20.0	1.6
Length of pectoral spine	23.0	20.1	30.3	25.4	2.0
Length of adipose-fin spine	10.5	8.7	15.2	12.2	1.4
Depth of caudal peduncle	17.9	15.5	19.4	17.6	0.8
Length of dorsal-fin base	20.6	17.1	23.4	19.8	1.3
Dorsal to adipose distance	23.2	15.8	24.3	19.3	1.5
Maximum cleithral width	31.3	24.5	31.3	26.9	1.1
Head length	41.1	35.9	44.4	40.8	1.8
Length of maxillary barbel	14.6	9.6	22.0	17.8	2.5
**Percents of Head Length**					
Head depth	98.3	83.0	102.3	92.5	4.0
Least interorbital distance	41.3	36.0	46.3	41.5	2.0
Horizontal orbit diameter	20.3	13.1	24.9	17.9	2.3
Snout length	31.4	26.5	35.8	30.8	1.9
Least internarial distance	18.6	18.4	28.8	21.7	1.8

SD = standard deviation.

Eye rounded, located mid-dorsally on lateral portion of head; orbit dorsally delimited by lateral ethmoid, frontal, sphenotic and antero-dorsal laminar expansion of infraorbital 1, ventrally by infraorbitals. Anterior and posterior nostrils close to each other, separated only by skin flap. Anterior nostril tubular. Posterior nostril near anterodorsal margin of orbit, separated from it by distance similar to naris diameter. Mouth small, subterminal, width approximately equal to bony orbit diameter. Maxillary barbel size ranging from moderate, not reaching anteroventral limit of gill opening, to large, slightly surpassing anteroventral limit of gill opening. Outer mental barbel generally slightly longer than maxillary barbel. Inner mental barbel fleshy, with base close to its counterpart. Small rounded papillae covering entire surface of all barbels, upper and lower lips, snout and isthmus.

Mesethmoid conspicuously short, clearly smaller than frontal length; anterior tip strongly reduced, smaller than 50% of bone length (see Britto, 2003: 123, character 1, state 1; fig 1B), posterior portion wide, entirely covered by thick layer of skin in most specimens; variably with small exposed area on its posteriormost portion. Narrow nasal, laterally curved, inner margin generally with poorly-developed laminar expansion; moderately-developed in some specimens; outer margin with laminar expansion ranging from clearly reduced to poorly developed; mesial border generally contacting only frontal; variably contacting both frontal and mesethmoid. Frontal elongated, relatively wide, width larger than half of entire length; anterior projection ranging from short, size smaller than nasal length, to long, size larger than nasal length. Frontal fontanel large, slender; posterior tip extension entering anterior margin of parieto-supraoccipital. Sphenotic roughly rectangular in shape, contacting parieto-supraoccipital dorsally, compound pterotic posteriorly, second infraorbital ventrally and frontal anteriorly. Compound pterotic roughly pipe-shaped, with posterodorsal portion contacting first lateral-line ossicle, and ventral margin contacting infraorbital 2, opercle and cleithrum. Parieto-supraoccipital wide, posterior process ranging from moderate to long in size, generally not contacting nuchal plate, contacting in single specimen (FML 7113, 31.1 mm SL); posterior portion of parieto-supraoccipital posterior process and anterior portion of nuchal plate generally covered by thick layer of skin; region between parieto-supraoccipital posterior process and nuchal plate with minute platelets in some specimens.

**Fig 4:**
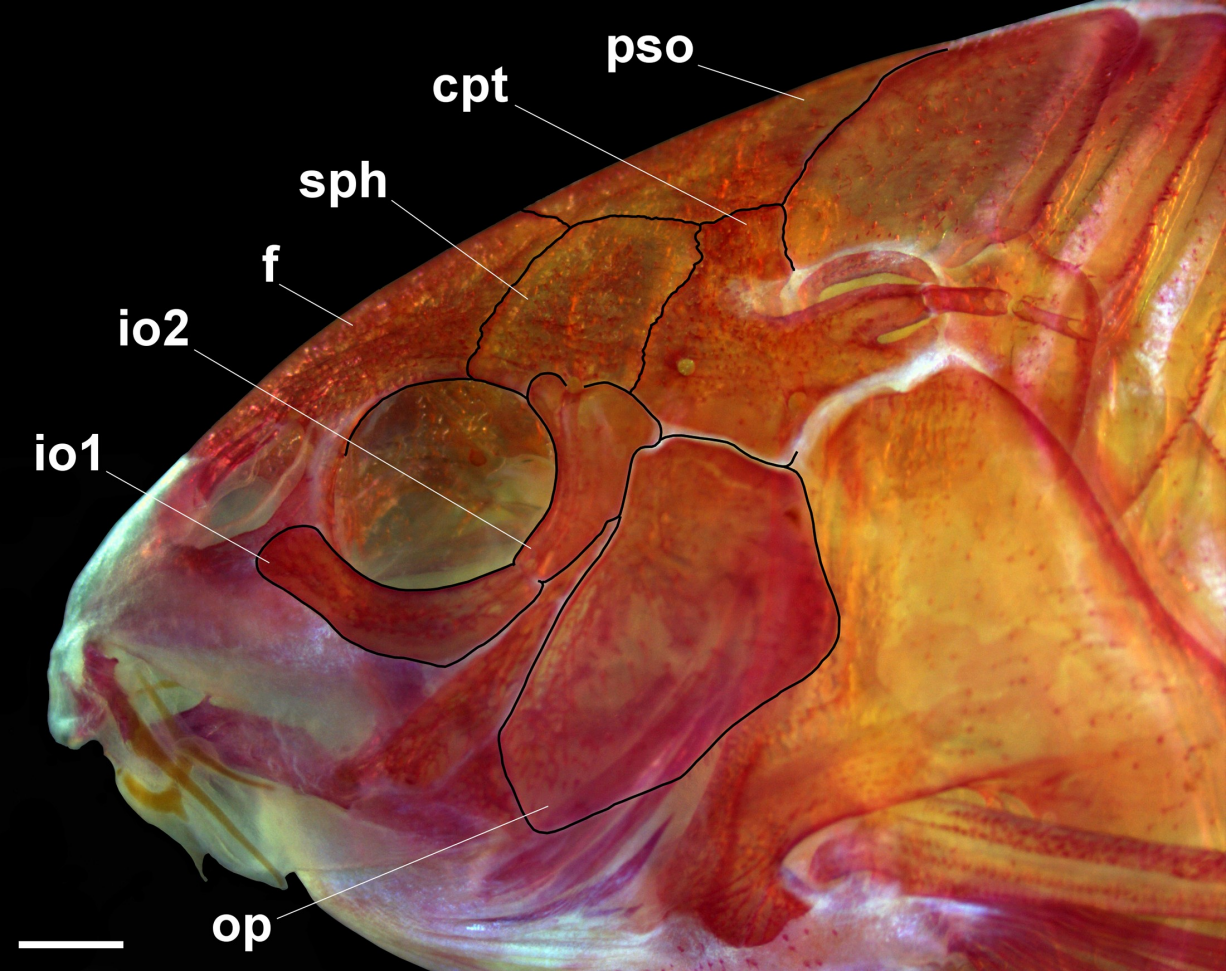
*Corydoras undulatus* skull. MCP 46587 (29.1 mm SL), left lateral view. Abbreviations: cpt = compound-pterotic, f = frontal, io1 = infraorbital 1, io2 = infraorbital 2, op = opercle, pso = parieto-supraoccipital, sph = sphenotic. Scale bars = 1.0 mm.

**Fig 5:**
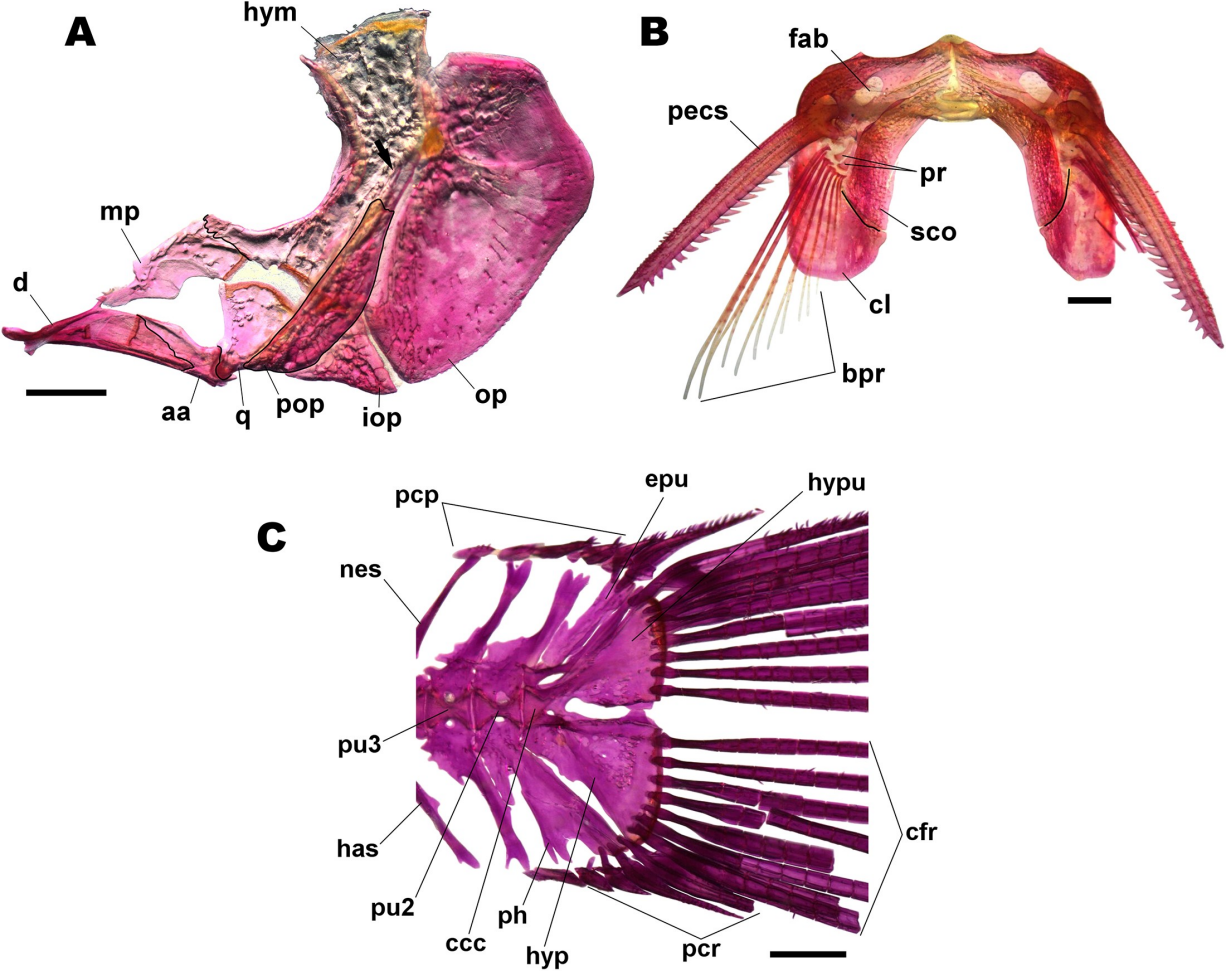
Dissected bones. UNICTIO 2600, 28.3 mm SL: (A) suspensorium and operculum of *Corydoras undulatus*. Lateral view. Abbreviations: aa = angle-articular, d = dentary, hym = hiomandibula, iop = interoperate, mp = metapterigoid, op = opercle, pop = preopercle, q = quadrate; (B) pectoral girdle of *Corydoras undulatus*. Ventral view. bpr = pectoral-fin ray, cl = cleithrum, fab = fossa of the abductor muscles of pectoral fin, pecs = pectoral-fin spine, pr = pectoral radials, sco = scapulocoracoid; (C) caudal-fin skeleton of *Corydoras undulatus*. Lateral view. ccc = compound ural centrum, cfr = principal rays, epu = epural, has = haemal spine, hyp = hypurals and parhypural, hypu = hypurals and uroneural, nes = neural spine, pcr = procurrent rays, ph = parhypural, pu2-3 = preural centrum. Scale bars = 1.0 mm.

Two laminar infraorbitals with minute odontodes; infraorbital 1 large, ventral laminar expansion generally ranging from poorly to moderately developed; few specimens with well-developed expansion; anterior portion with laminar expansion ranging from poorly developed, slightly surpassing posterior margin of nasal capsule, to moderately developed, reaching to middle of nasal capsule; inner laminar expansion ranging from strongly reduced to poorly developed; small portions of external surface covered by thick layer of skin (Fig 4); infraorbital 2 small, relatively wide; posterior laminar expansion well developed; inner laminar expansion ranging from poorly to well developed; posteroventral margin contacting posterodorsal ridge of hyomandibula, dorsal tip contacting sphenotic and compound pterotic; small portions of external surface covered by thick layer of skin (Fig 5A). Posterodorsal ridge of hyomandibula close to its articulation with opercle conspicuously slender, exposed; dorsal ridge of hyomandibula between compound pterotic and opercle covered by thick layer of skin or by posterodorsal laminar expansion of infraorbital 2; exposed areas bearing small odontodes. Interopercle almost entirely exposed, with anterior tip covered by thick layer of skin; somewhat triangular, anterior projection ranging from moderately to well developed. Preopercle elongated, relatively slender; minute odontodes on external surface. Opercle dorsoventrally elongated, relatively compact, width slightly larger than half of its length; free margin convex; posterodorsal region generally with slightly concave area; without serrations and covered by small odontodes; some portions of bony distal margin generally irregular.

Four branchiostegal rays decreasing in size posteriorly. Hypobranchial 2 somewhat triangular, tip ossified and directed towards anterior portion, posterior margin cartilaginous; ossified portion well developed, about twice size of cartilaginous portion. Five ceratobranchials with expansions increasing posteriorly; ceratobranchial 1 with small process on anterior margin of mesial portion; process strongly reduced in some specimens; ceratobranchial 3 with continuous postero-lateral margin; ceratobranchial 5 toothed on postero-dorsal surface, 35 to 38 (2) teeth aligned in one row. Four epibranchials with similar size; epibranchial 2 slightly larger, with small pointed process on laminar expansion of posterior margin; epibranchial 3 with somewhat triangular uncinate process on laminar expansion of posterior margin. Two wide pharyngobranchials (3 and 4), pharyngobranchial 3 with triangular laminar expansion on posterior margin; expansion with notches in some specimens. Upper tooth plate oval; 37 to 43 (2) teeth aligned in two rows on postero-ventral surface.

Lateral-line canal entering neurocranium through compound pterotic, generally branching twice before entering sphenotic: pterotic with single pore; preoperculomandibular branch conspicuously reduced, with a single pore opening close to postotic main canal; single specimen (MCP 46587, 29.1 mm SL) with third branch just posterior to pterotic branch. Sensory canal continuing through compound pterotic, entering sphenotic as temporal canal, which splits into two branches: one branch giving rise to infraorbital canal, other branch entering frontal through supraorbital canal, both with single pore. Supraorbital canal branched, running through nasal bone. Epiphyseal branch conspicuously reduced; pore opening close to supraorbital main canal, directed towards frontal fontanel. Nasal canal with three openings, first on posterior edge, second on posterolateral portion, generally fused with first pore, and third on anterior edge. Infraorbital canal running through entire second infraorbital, extending to infraorbital 1 and opening into two or three pores. Preoperculomandibular branch giving rise to preoperculo-mandibular canal, which runs through entire preopercle with three openings, leading to pores 3, 4, and 5, respectively.

Dorsal fin roughly triangular, located just posterior to third dorsolateral body plate. Dorsal-fin rays II,8* (49), posterior margin of dorsal-fin spine with one to five poorly-developed serrations directed towards tip of spine; serrations generally restricted to distal portion of spine. Nuchal plate ranging from moderately developed to well developed; almost entirely exposed, with minute odontodes; spinelet short; spine ranging from moderately developed, adpressed distal tip slightly surpassing middle portion of dorsal-fin base, to relatively well developed, adpressed distal tip slightly surpassing posterior origin of dorsal-fin base; anterior margin with small odontodes. Pectoral fin roughly triangular, its origin just posterior to gill opening. Pectoral-fin rays I,8* (49); posterior margin of pectoral spine with 16 to 24 conical serrations along almost its entire length; most serrations strongly developed and directed towards origin of spine; proximal and/or distal edge of spine variably with less-developed serrations; some serrations perpendicularly directed or directed towards tip of spine in some specimens; bifid serrations in some specimens (Fig 5B). Anteroventral portion of cleithrum exposed; posterolateral portion of scapulocoracoid moderately developed, exposed, with anterior portion poorly to moderately expanded, not contacting anteroventral portion of cleithrum; minute odontodes sparse on exposed areas. Pelvic fin oblong; located just below second ventrolateral body plate, and at vertical through first or second branched dorsal-fin rays. Pelvic-fin rays i,5* (49). Adipose fin roughly triangular, separated from base of last dorsal-fin ray by generally six dorsolateral body plates. Anal fin somewhat triangular, located just posterior to 11^th^ or 12^th^ ventrolateral body plates, and at vertical through region of preadipose platelets or anterior portion of adipose-fin spine. Anal-fin rays ii,6* (49). Caudal-fin rays i,12,i* (49), generally with four dorsal and ventral procurrent rays; bilobed, dorsal and ventral lobes generally with similar size (Fig 5C).

Generally, two laterosensory canals on trunk; first ossicle tubular, second ossicle laminar; second ossicle lacking canal in one side of a single specimen (MCP 46587, 29.1 mm SL). Body plates with minute odontodes scattered over exposed area, a conspicuous line of odontodes confined on posterior margins; dorsolateral body plates 22* (135), 21 (29); ventrolateral body plates 19 (20), 20* (143); dorsolateral body plates along dorsal-fin base 7* (164); dorsolateral body plates between adipose- and caudal-fin 6* (164); preadipose platelets 2 (32), 3 (7), 4* (125); small platelets covering base of caudal-fin rays; small platelets disposed dorsally and ventrally between junctions of lateral plates on posterior portion of caudal peduncle. Anterior margin of orbit, above region of junction between frontal and lateral ethmoid, generally with small platelets bearing odontodes. Ventral surface of trunk with small platelets, generally more concentrated on regions of pectoral and pelvic girdles.

Vertebral count 21 (4), 22 (1); ribs 6 (4), 7 (1) first pair conspicuously large; parapophysis of complex vertebra ranging from poorly to moderately developed.

### Color in alcohol

Overall color of body in Fig 3. Background color of body brownish yellow, with top of head dark brown. Dorsal and lateral surfaces of head densely covered by dark brown or black chromatophores, variably forming a marbled pattern of blotches; cleithrum, external surfaces of upper and lower lips, maxillary and outer mental barbels, and ventrolateral portion of snout with scattered dark brown or black chromatophores; region of isthmus just posterior to mouth and inner mental barbel variably with dark brown or black chromatophores. Flanks with relatively large dark brown or black markings, generally forming marbled or somewhat anastomosed pattern; blotches variably forming up three irregular and/or intermittent longitudinal bands; first band, if present, along dorsolateral body plates, second, if present, along midline of flank, and third, if present, along ventrolateral body plates; third band more regular and continuous in some specimens. Ventral portion of ventrolateral body plates generally with sparse dark brown or black chromatophores. Dorsal fin covered by dark brown or black chromatophores, generally more concentrated on its dorsal half; variably forming irregular diffuse dark blotches; somewhat longitudinally aligned blotches in some specimens. Pectoral fin with dark brown or black chromatophores, generally restricted to spine and rays, variably forming diffuse dark blotches. Pelvic, adipose and anal fins with dark brown or black chromatophores, forming dark blotches in some specimens. Caudal fin with dark brown or black chromatophores, generally restricted to rays; often forming dark blotches transversely aligned in irregular bars.

### Color in life

**Fig 6:**
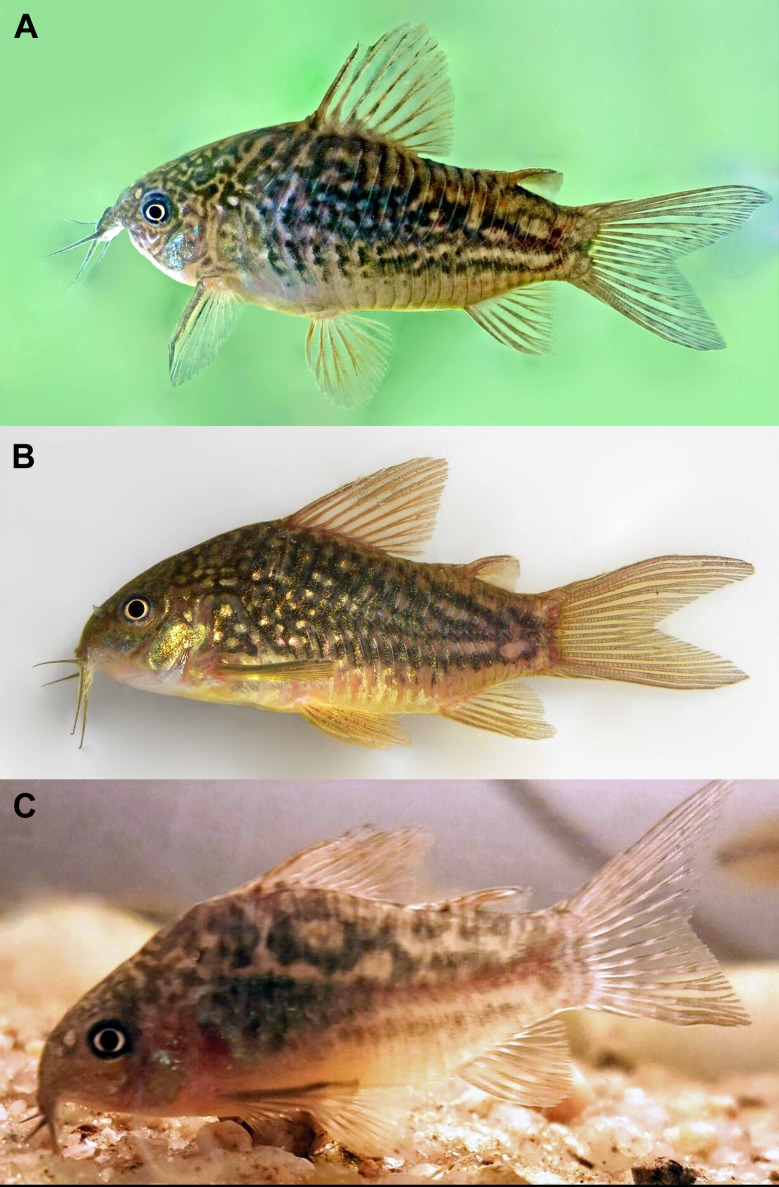
Color in life of *Corydoras undulatus* in left lateral view. (A) IBIGEO-I 452, 43.1 mm SL, Yapeyu (29°28'14.30"S 56°48'59.41"W). (B) MHNM 4000, 32.5 mm SL, Rincón de Franquia, Bella Unión, Artigas, Uruguay (30°11'45.75"S 57°37'8.40"W), (Photograph: Wilson Sebastián Serra Alanis); (C) IBIGEO-I 451, 37.4 mm SL, San Lorenzo stream, Paraná River basin, at Entre Ríos Province in Argentina (Photograph: Roberto Toval).

Similar to preserved specimens, but with greyish yellow background color of body. Greenish yellow iridescent coloration on body (Fig 6).

### Ontogenetic color pattern changes

**Fig 7:**
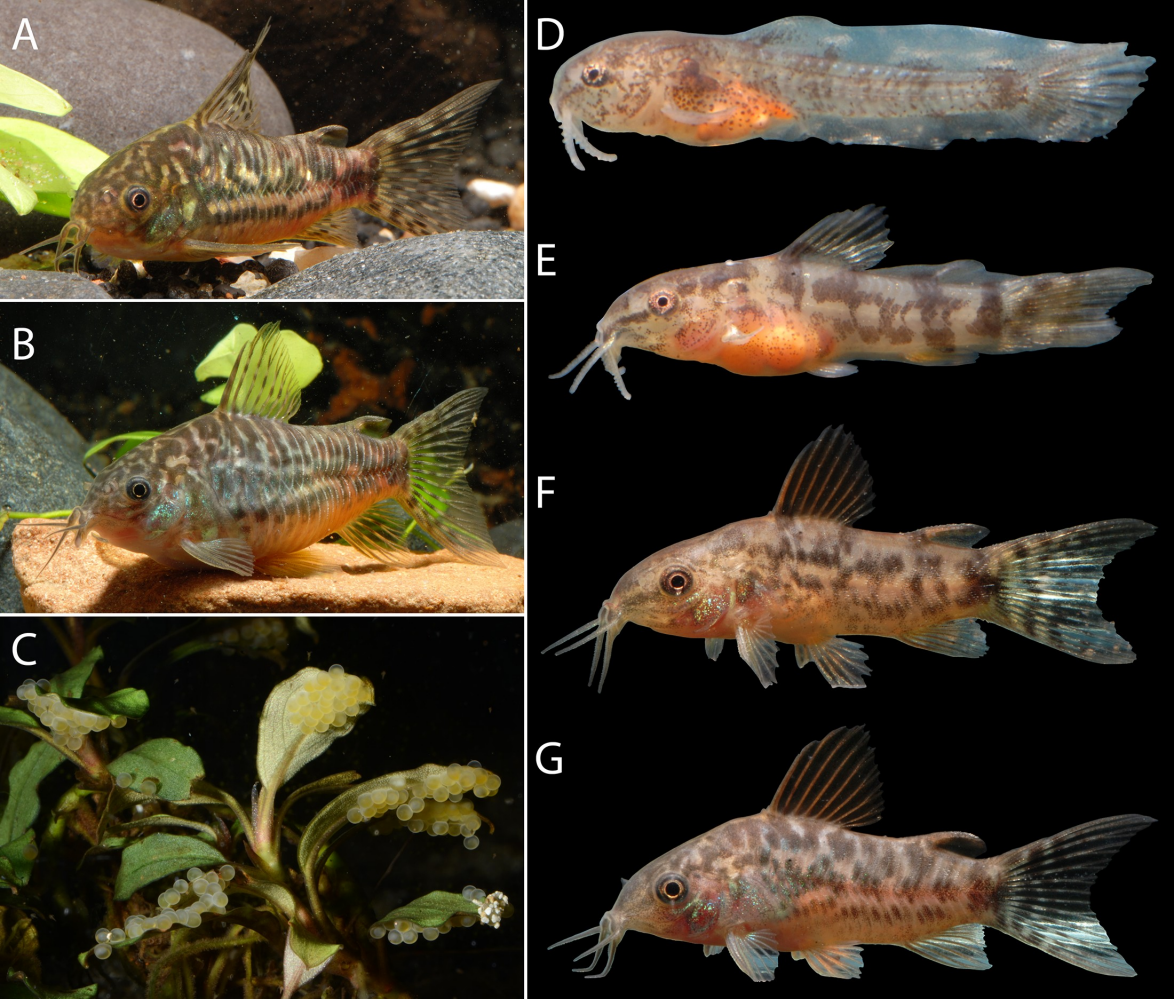
Ontogenetic variation of color pattern of *Corydoras undulatus*. Left lateral view. (A) male, approx. 40 mm TL; (B) female, approx. 45 mm TL; (C) eggs adhered to submerged vegetation, about 1.5 mm in diameter; (D) 7 days post hatching (PH), approx. 7 mm TL; (E) 2 weeks PH, approx. 11 mm TL; (F) 4 weeks, approx. 15 mm TL; (G) 3 months, approx. 24 mm TL. (Photograph: Hans Evers).

Juvenil fish, after 7 days of birth without lateral stripe on the head behind the eye; after one month of post hatching (aprox. 8.5 mm SL) with oval blotches on dorsal plates series and posterior ventral plates; caudal fin with dark blotches transversely aligned in irregular bars. (Fig 7).

### Sexual dimorphism

**Fig 8:**
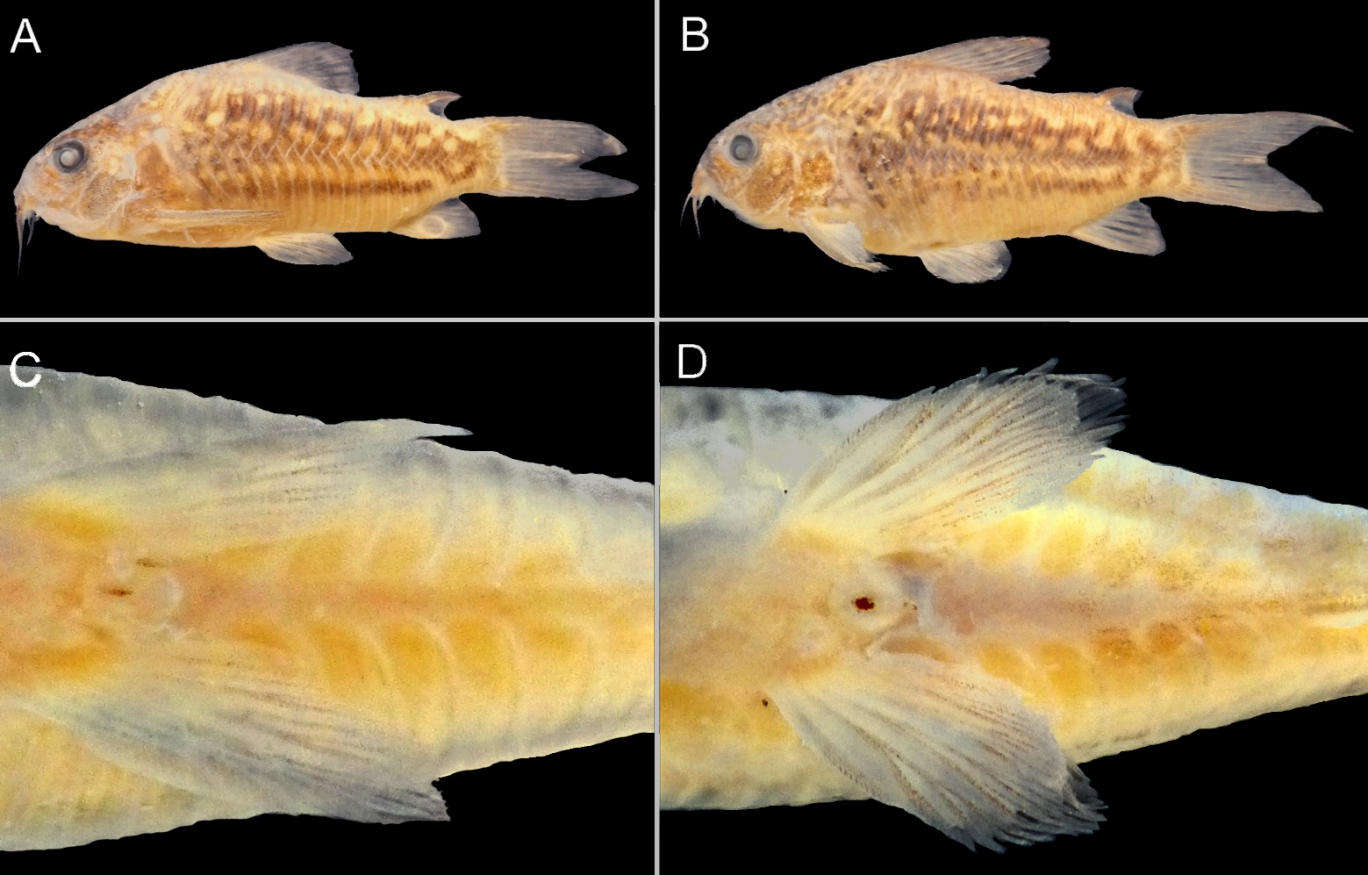
Photo of preserved specimens of *Corydoras undulatus*. IBIGEO-I 452, 43.1 mm SL, Yapeyu (29°28'14.30"S 56°48'59.41"W): (A) male in lateral view; (B) female in lateral view; (C) pelvic fin and urogenital papillae of male in ventral view; (D) pelvic fin and urogenital papillae of female in ventral view.

Males specimens exhibit a genital papillae with a tubular shape as most species of Corydoradinae and most species of lineage 6 [22]. Males are generally smaller than females with a darker color pattern tending to form clear longitudinal irregular lines on the laterals of the body *vs*. females presenting a more diffuse color pattern. Also, males present more elongated and pointed pelvic fins than females. In females the ventral plates between the pelvic and anal fins are more separated medially (Fig 8).

### Geographic distribution

**Fig 9:**
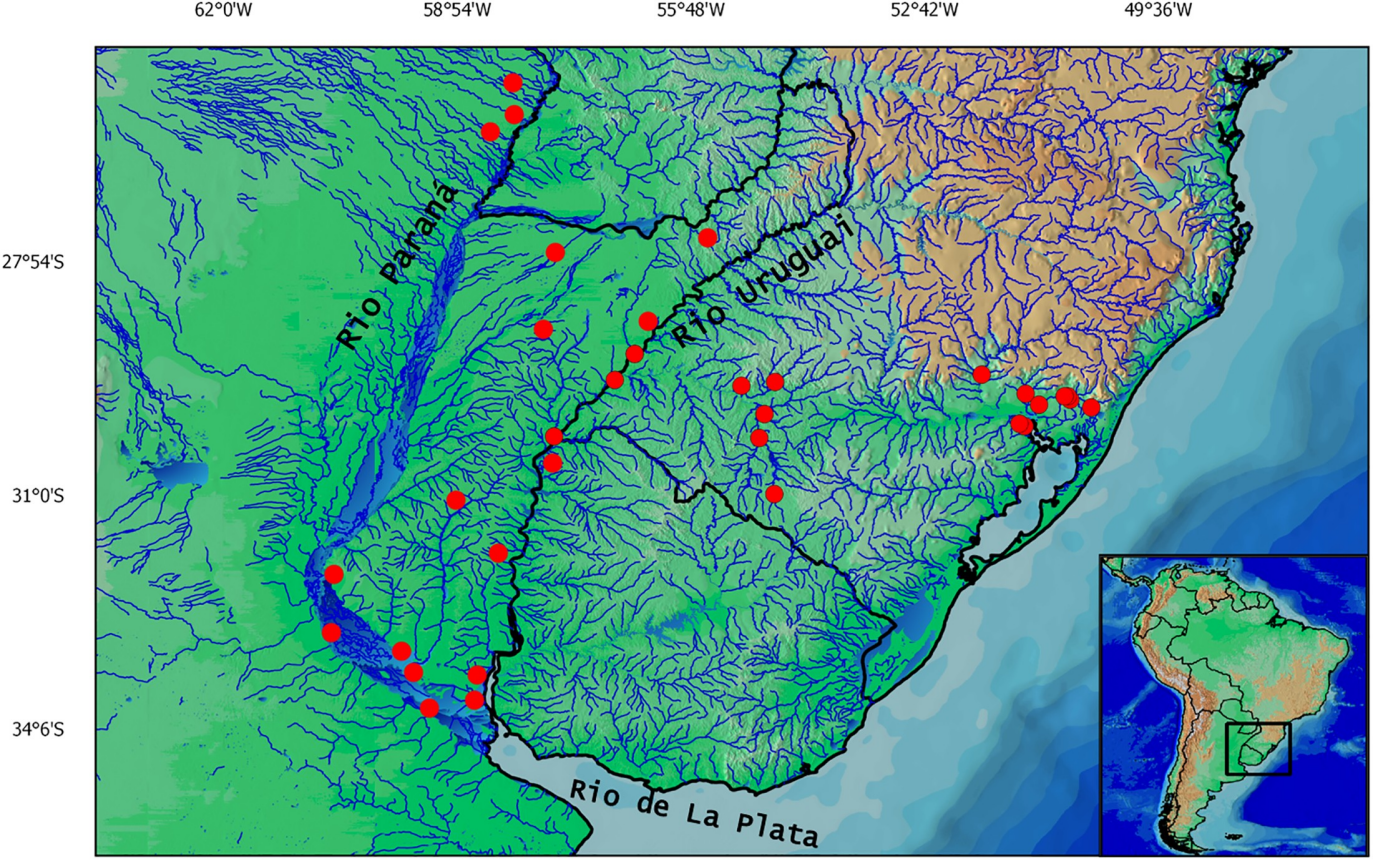
Map with the geographical distribution of *Corydoras undulatus*. Each symbol (red circles) may represent more than one locality.

*Corydoras undulatus* is known from the Paraguay River basin in Paraguay and Argentina, Paraná River basin in Argentina, in the Uruguay river basin in Uruguay, Argentina and Brazil and the Laguna dos Patos in Rio Grande do Sul, Brazil (Fig 9).

### Principal Component Analysis (PCA)

**Fig 10:**
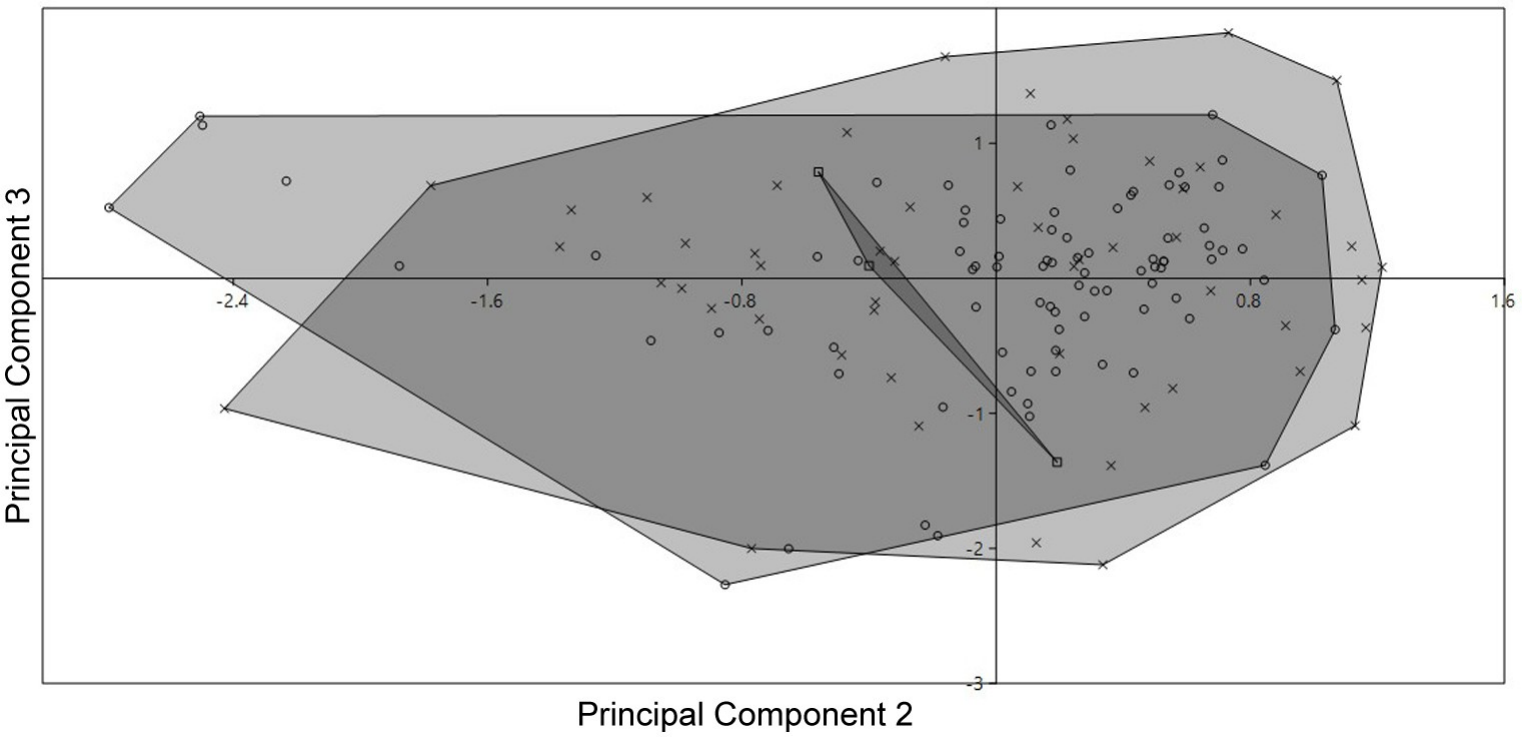
Principal Component Analysis (PCA). Plots of factor scores of principal component analysis of three populations of *Corydoras undulatus*. Square from río Paraná basin; circle from Uruguay basin; cross from Laguna dos Patos basin.

No differences were found among the evaluated populations of *Corydoras undulatus* with the analyzed variables indicating no morphometric differences among them (Fig 10).

### Ecological notes

**Fig 11:**
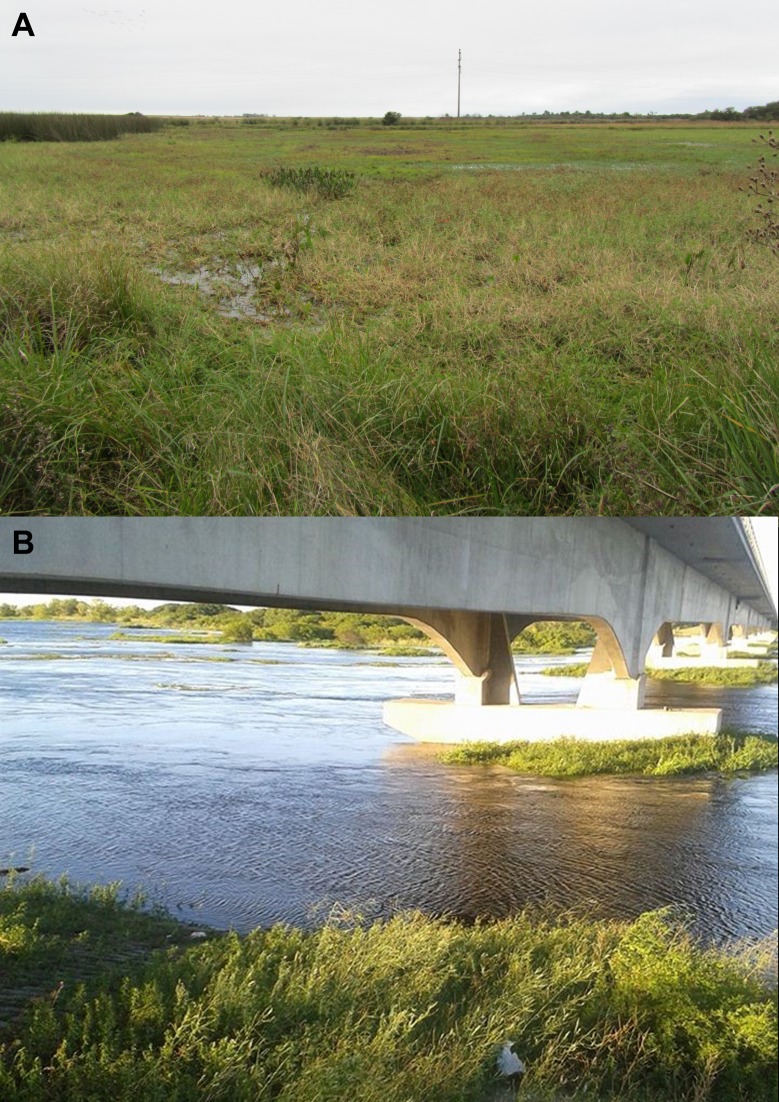
Habitat of *Corydoras unduluatus*. (A) Near Yapeyú, Corrientes, Argentina where this species is particularly abundant in habitats with low water flow and abundant aquatic macrophytes. (B) *Corydoras undulatus* capture site in San Lorenzo stream, affluent of Paraná River, Province of Entre Ríos, Argentina, (32°48'2.28"S 60° 29'56.63"W) (Photograph: Roberto Toval).

*Corydoras undulatus* is generally found in vegetated environments (Fig 11A) where it is more abundant, but can also be eventually found in low abundance in open waters in rivers (Fig 11B).

### Remarks

As explained by Tencatt & Evers [23], the color pattern of the Corydoradinae considerably change from their hatching till the adult stage. *Corydoras undulatus* is well known among fishkeeping hobbyists, and it was successfully bred under aquarium conditions by Hans-George Evers, who documented *C*. *undulatus* development during its growth, including photos of eggs deposited in an aquatic macrophyte. In the aquarium hobby, putative new species of Corydoradinae are generally inserted in a widely-known code-system with the intention of avoiding the creation of *nomina nuda*. (see Tencatt & Evers, 2016). Although some of the coded species strongly resembles *C*. *undulatus*, such as C88, said to be from Mato Grosso, Brazil, C132, with unknown locality, no specimen related to these codes and/or available locality could be examined herein, making it impossible to undoubtedly attribute these speciemens to *C*. *undulatus*. However, another extremely similar coded species, CW22, is said to be from the Rio Paraguay, Argentina, which matches one of the localities of our examined material. Considering the available collecting data plus photos of CW22 (Fig 7), it seems that these specimens indeed can be determined as *C*. *undulatus*.

### Material examined

Type specimens. *Corydoras undulatus*: Argentina: BMNH 1912.7.10.5, holotype, 41.8 mm SL, La Plata. BMNH 1909.9.28.3–4, 2, paratypes, 25.3–26.1 mm SL, “La Plata”.

Non-type specimens. *Corydoras undulatus*: Brazil, Rio Grande do Sul. -MCP 8418, 1, 23.5 mm SL, Rio Santa Maria, Dom Pedrito, 30°59'0.00"S 54°42'0.00"W; MCP 41943, 2, 35.7–46.8 mm SL, Wetland drainage of Rio Cacequi, Cacequi, 29°55'23.00"S 54°49'52.00"W; MCP 23092, 4, 31.0–35.6 mm SL, Rio Inhacunda, São Francisco de Assis, 29°32'51.00"S 55° 8'11.01"W; MCP 46587, 6, 26.3–33.7 mm SL (1 c&s 38.9 mm SL), Uruguaiana, 29°29'51.00"S 54°41'24.00"W; MCP 30655, 2, 19.9–20.4 mm SL, Biological Reserve Wetland São Donato, Itaqui, 29°7'31.08"S 56°33'11.16"W; MCP 30654, 4, 15.7–22.5 mm SL, Biological Reserve Wetland São Donato, Itaqui, 29° 7'31.08"S 56°33'11.16"W; MCP 13954, 3, 21.1–38.9 mm SL (1 c&s 29.5 mm SL), stream affluent Rio Jacuí, El dorado do Sul, 30° 2'60.00"S 51°27'0.00"W; MCP 28987, 9, 28.8–38.2 mm SL (1 c&s 32.5 mm SL), Arroio passo dos carros, Eldorado do Sul, 30° 5'0.00"S 51°23'0.01"W; MCP 17317, 3, 25.5–29.3 mm SL, Arroio da Porteira, Arroio dos Ratos, 29°23'60.00"S 51°57'0.00"W; MCP 15770, 2, 33.1–38.2 mm SL, Stream on the road between BR 290 and Guaíba city, El dorado do Sul, 30° 2'60.00"S 51°21'59.99"W; MCP 13953, 4, 28.4–35.5 mm SL, Stream affluent of Lago Guaíba, Eldorado do Sul, 30°2'60.00"S 51°23'0.01"W; MCP 14671, 6, 27.3–36.1 mm SL, Drainage of Rio dos Sinos, Rio Jacuí basin, Caraá, 29°46'60.00"S 50°25'0.00"W; MCP 17316, 2, 34.3–34.8 mm SL, Arroio Porteira, Arroio dos Ratos, 29°23'60.00"S 51°57'0.00"W; MCP 15769, 2, 28.5–34.0 mm SL, Creek 2 km from West of the BR-116 junction with the BR-290, Eldorado do Sul, 30° 2'60.00"S 51°20'59.99"W; UNICTIO 2598, 3, 34.1–36.3 mm SL, Arroio Entrepelado, Taquara, 29°43'54.00"S 50°46'33.20"W; UNICTIO 2599, 3, 31.4–37.9 mm SL (1 c&s 31.7 mm SL), Rio Cai basin, Capela de Santana, 51°22'5.00"S 51°22'5.00"W; UNICTIO 2600, 52, 22.4–30.2 mm SL (3 c&s 25.4–28.3 mm SL), Rio Ibicuí da Armada, Rosário do Sul, 30°14'24.00"S 54°53'60.00"W; MZU 846, 3, 37.6–39.3 mm SL, Santa Cristina do Pinhal, 29°41'11.12"S 50°51'3.16"W; MZU 1509, 1, 36.5 mm SL, Aquaculture facility Sapucaia, Sapucaia do Sul, 29°47'56.61"S 51°11'24.68"W; MZU 1250, 6, 36.0–45.1 mm SL, near of the Rio dos Sinos, Taquara, 29°41'11.12"S 50°51'3.16"W; MZU 1230, 15, 35.66–45.45 mm SL (2 c&s 37.74–39.8 mm SL), near of the Rio dos Sinos, Taquara, 29°41'11.12"S 50°51'3.16"W; MZU 614, 5, 24.2–30.0 mm SL, Arroio Ponte Guarda Velha, Santo Antônio da Patrulha, 29°50'7.77"S 50°29'51.02"W; CFA-IC-1348, 1, Quaraí creek, Paso da Guarda.

Uruguay: -MNHN 8459, 5, 32.1–37.7 mm SL (1c&s 33.5 mm SL), Rio Uruguay, Franquía, Exit from marginal lagoom, Artigas, 30°13'9.38"S 57°37'20.32"W; MHNM 4000, 1, 32.5 mm SL, Rincón de Franquia, Bella Unión, Artigas, 30°11'45.75"S 57°37'8.40"W.

Argentina: Corrientes: -FML 7113, 6, 28.1–32.2 mm SL (1 c&s 31.1 mm SL), Yapeyu, 29°28'14.30"S 56°48'59.41"W; IBIGEO-I 452, 4, 35.9–43.1 mm SL, Yapeyu, 29°28'14.30"S 56°48'59.41"W. Entre Ríos: IBIGEO-I 451, 2, 36.0–37.4 mm SL (1 c&s 37.4 mm SL), Arroyo San Lorenzo affluent of the Paraná River, 32°48’2.28”S 60°29’56.63”W; CFA-IC-1015, 10, Bañado Parió—Pá and Route provincia 94, Santo Tomé; CFA-IC-4641, 1, Ayuí Grande river, Mercedes department, 28° 57'10,00”S 57°39'19,40”W; CFA-IC-3749, 4, Lucas creek overflow, 03/09/2004; CFA-IC-4189, 1,, Concepción stream. CFA-IC-4640, 4, Brasilero stream, old house, Villa Paranacito, 33°46'57,40”S 58°32'52,6”W; CFA-IC-4822, 1, Brasilero stream, old house, Islas del Ibicuy, Villa Paranacito, 33°46’57,4”S 58°32’52,6”W; CFA-IC-6447, 1, Brasilero stream, Paraná river basin, Chozas, Isla de Ibicuy, Villa Paranacito, 33°47´7,5”S 58° 33´16,5”W. Formosa: CFA-IC-1316, 3, Gral. Sanchez, stream in the road from Gral. Sanchez to Colonia Pastoril; FML 2035,38, Riacho Formosa, Formosa Province, 26° 10' S 58° 09' W; CFA-IC-7098, 1, Estero 1, road to Boedo, Formosa, Paraguay river basin, 26°3´24,6”S 58°25´44,0”W. Misiones: CFA-IC-11537, 5, Chirimay Miní stream, under the bridge of RP 105 to San José a Apóstoles, near the camping area, Apóstoles department, Uruguay river basin, 27° 52'S 55° 48'W.

### Conservation status

The species is widely distributed and do not seem to present immediate concerns with respect to its conservation status. Nevertheless, the area of occurrence is widely impacted by agriculture and habitat modifications such as canalizations that may affect those populations.

## Discussion

According to Regan, the type locality of *C*. *undulatus* is “La Plata”, without further details. However, “La Plata” could refer to the city of La Plata in Buenos Aires or to the La Plata basin. Specimens of *C*. *undulatus* were searched in main Argentinean collections (MACN, MLP, FA, IBIGEO, FML), and it was not possible to find any lot of *Corydoras undulatus* from La Plata city, neither from the left bank of La Plata River basin or the Pre-Delta of Paraná area in any ichthyological collection. It is worth noting that in this area there have been ichthyologists since the late XIX century, and in La Plata city itself. Additionally, the specimens in which the description was based were donated by JP Arnold and WG Wolterstorff. Neither these people are known to have been in South America. Furthermore, Johann Paul Arnold (1869–1952) was a German aquarium hobbyist who forwarded numerous fish specimens taken from commercial aquarium imports to Germany to ichthyologist for identification [24]. Willy Georg Wolterstorff (1864–1943) was a German paleontologist and herpetologist [25]. Therefore, those specimens were more probably imported as aquarium fish and donated to Regan and the type locality should be considered as uncertain, probably from somewhere in “La Plata” basin. Most likely places are those from southern Entre Ríos where this species is commonly found and are close localities to the Port of Buenos Aires where is the most likely place where those fish were shipped to Europe.

The comparative analysis of the linear measures among the different basins did not show significant differences, as well as the principal component analysis (PCA) (Fig 10). Thus, based on these results plus meristic comparisons and osteological analysis we conclude that there are no evidences to consider any of these populations as a different taxon.

After revising the pictures of *C*. *undulatus* corresponding to lots deposited in the Swedish Museum of Natural History from the Paraguay River drainage in Paraguay, we conclude that those specimens probably correspond to *C*. *undulatus* [catalog numbers: NRM 23550, NRM 31026, NRM 31483, NRM 33556, NRM 45056, NRM 45060 and NRM 65433]. Here we also confirm the presence of *C*. *undulatus* in Uruguay Republic based on voucher materials for the first time.

Alexandrou *et al*. [26], obtained *Corydoras sensu lato* in their phylogenetic analysis as a paraphyletic group composed by seven monophyletic clades. As discussed by Tencatt & Pavanelli [27], one of the most conspicuous groups within *Corydoras* comprises the species of the lineages 4 and 5, which is basically conformed by Nijssen & Isbrücker’s (1998) “*Corydoras elegans* group” species, including *C*. *undulatus*, plus *C*. *bilineatus*, *C*. *gracilis*, *C*. *napoensis*, *C*. *nijsseni* and some unidentified species. Even though Nijssen & Isbrücker [4] have assigned *C*. *latus* to the *C*. *elegans* group (Nijssen & Isbrücker, 1980), this name is often erroneously attributed to female specimens of *C*. *pantanalensis* in several museums and fish collections (LFCT pers. obs.). One of the most reasonable explanations for this can be the work published by Knaack [28], in which he discussed the identity of *C*. *aurofrenatus* and *C*. *latus*. Knaack ([29]: 19) argued that the serration pattern of the pectoral-fin spine displayed by *C*. *latus* holotype is compatible to what he called the “*Corydoras reticulatus* group”, which, according to this author, comprises *C*. *reticulatus*, *C*. *sodalis*, *C*. *geryi* and *C*. *pantanalensis* (see Knaack [29]: 51).

**Fig 12:**
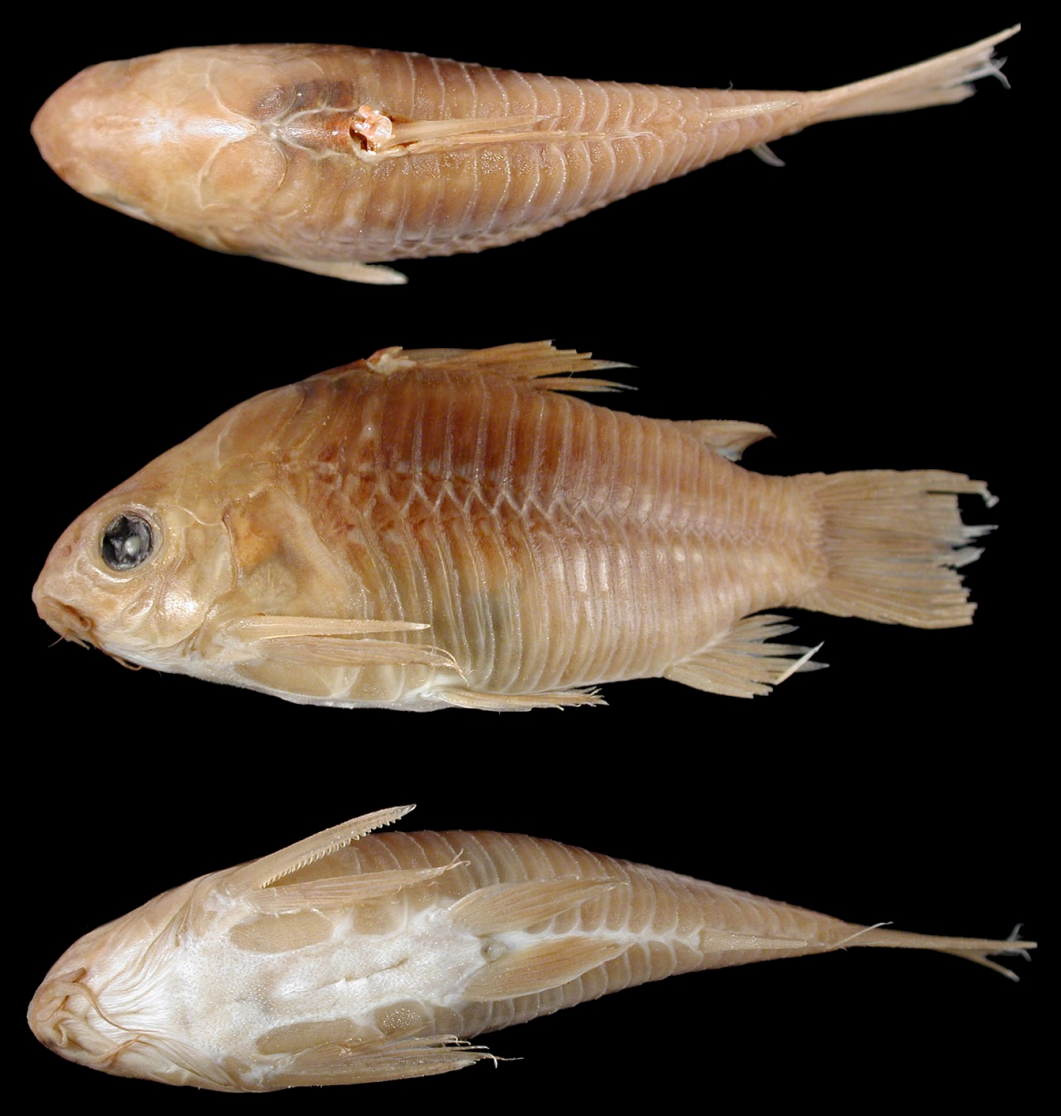
*Corydoras latus*, holotype. CAS 36452 [ex IU 17278], 41.5 mm SL, Lago Rojo Agua, Río Beni basin, Upper Amazon system, Beni, Bolivia. coll. N. E. Pearson, 1921. Lateral, dorsal and ventral view.

The close relationship between the species of Knaack’s [29] “*Corydoras reticulatus* group” was posteriorly corroborated by Alexandrou *et al*. ([26]: 3, supplementary fig. 2), which recovered these four species forming a small monophyletic clade within the lineage 8. Despite of that, Knaack’s [28] argumentation regarding the serration pattern of *C*. *latus* holotype and his “*Corydoras reticulatus* group” could be refuted herein. In a closer look, the serrations along posterior margin of the pectoral-fin spine in *C*. *latus* holotype are strongly-developed conical serrations, mostly directed towards origin of spine. Such pattern is present only in *Aspidoras* (Tencatt [30]: 185, fig. 51b), *Scleromystax* (Britto *et al*. [31]: e150158, fig. 5) and *Corydoras* from the lineages 1 ([19]: 434, fig. 2c), 4 ([26]: 291, fig. 4) and 5 (Fig 5B), whereas the typical pectoral-fin spine serration pattern of the lineage 8 species is characterized by the presence of moderately-developed laminar serrations, mostly directed towards origin of spine ([19]: e150063, fig. 3c), and which can be used to distinguish the species within this lineage from all others congeners. In addition to the pectoral-fin spine serration pattern, the analysis of the available photos of the holotype of *C*. *latus* (Fig 12; Knaack [28]: 21) revealed the presence of a conspicuously short and rounded snout, readily distinguishing it from the congeners of the lineages 1 and 8 (*vs*. snout well developed and pointed, generally with a slightly concave lateral profile; nearly straight in some specimens in lineage 1 species; snout generally well developed, pointed and straight, variably with a slightly concave lateral profile; some specimens with moderately developed and/or rounded snout in lineage 8 species).

**Fig 13:**
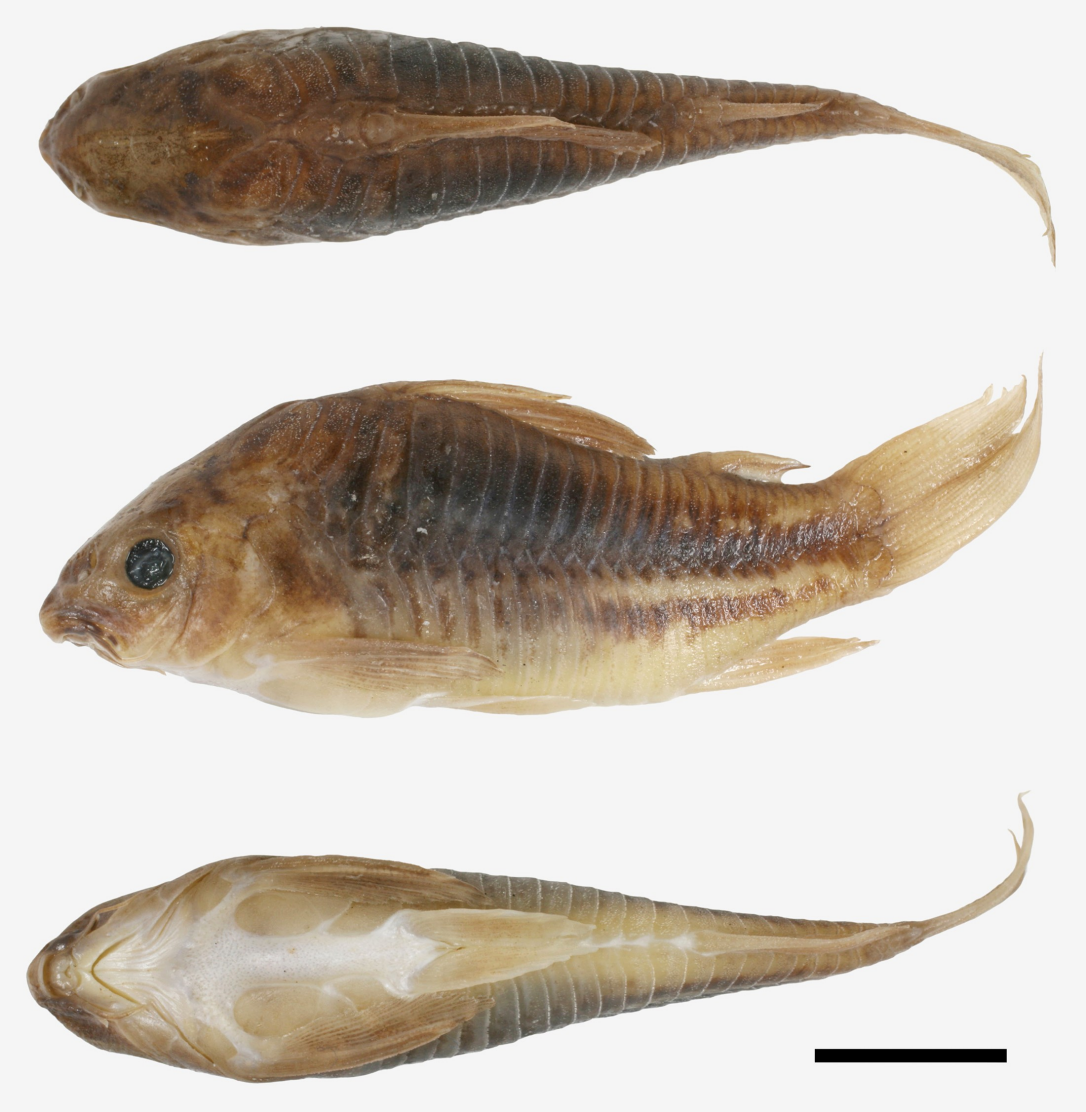
*Corydoras bilineatus*, paratype. ZMB 33297, 45.3 mm SL, Chené, Santiesteban, Santa Cruz Dept., Bolivia. coll. J. Knaack, 2001. Lateral, dorsal and ventral view. Scale bars = 10 mm. (Photograph and credit by Edda Aβel, Museum für Naturkunde Berlin).

Although the correlation between *C*. *latus* and the species from the “*Corydoras elegans* group” can be promptly verified even through the analysis of photographs of its holotype, the recognition of its identity as species is still unclear. This species is currently known only from its holotype, which seems to be a large-sized female (about 40.0 mm SL with absence of visible genital papillae). As presented in previous publications (Nijssen & Isbrücker [32]; Knaack [28, 29]), the species from this group often present a wide variation range in color pattern during their development and also between sexes. Therefore, only through the analysis of material containing juvenile, adult, male and female specimens it will be possible to clearly recognize *C*. *latus*. Interestingly, the color pattern of the holotype of *C*. *latus* (Fig 12) resembles the one displayed by females of *C*. *bilineatus* (Fig 13), both species from the rio Madeira basin, Bolivia. However, no specimen of *C*. *latus* could be examined herein, except by the photographs of its holotype, and, by this reason, no changes regarding its taxonomic status shall be suggested until further analysis.

### Comparative material examined

In addition to the material listed by Espíndola *et al*. [33], the following specimens were analyzed. **Brazil**, Amazonas. *Corydoras elegans*: BMNH 1889.11.14.55–58, 4, paralectotypes, 24.6–36.1 mm SL; USNM 41531, 2 paralectotypes, 26.7–28.2 mm SL; USNM 41532, 2 paralectotypes, 32.5–35.0 mm SL; USNM 120252, 6 paralectotypes, 26.2–31.1 mm SL; USNM 216716, 10 paralectotypes, 36.3–43.3 mm SL, Amazon River at Tefé *Corydoras gracilis*: BMNH 1976.4.27.143, 1, paratype, 17.9 mm SL, Rio Jauna (= Rio Juma) at Trans-Amazonica high way. USNM 216074, 1, paratype, 19.2 mm SL, same data as previous lot. **Ecuador**, Napo. *Corydoras napoensis*: USNM 270358, 2 paratypes, 26.7–28.3 mm SL, Lagartococha, northern tributary of the Río Aguarico.
